# Risk prediction model for progression of type 2 diabetic nephropathy with and without metabolic syndrome: a retrospective cohort study

**DOI:** 10.3389/fendo.2025.1592180

**Published:** 2025-07-30

**Authors:** Yuan Fang, Siyi Rao, Yongjie Zhuo, Jiaqun Lin, Xiaohong Zhang, Jianxin Wan

**Affiliations:** ^1^ Department of Nephrology, Blood Purification Research Center, The First Affiliated Hospital, Fujian Medical University, Fuzhou, China; ^2^ Fujian Clinical Research Center for Metabolic Chronic Kidney Disease, The First Affiliated Hospital, Fujian Medical University, Fuzhou, China; ^3^ Department of Nephrology, National Regional Medical Center, Binhai Campus of the First Affiliated Hospital, Fujian Medical University, Fuzhou, China

**Keywords:** type 2 diabetic nephropathy, metabolic syndrome, clinicopathologic features, prognosis, prediction model

## Abstract

**Objectives:**

To construct a risk prediction model for type 2 diabetic nephropathy (T2DN) progression in patients with and without metabolic syndrome (MetS).

**Methods:**

In this retrospective study, we enrolled 130 T2DN patients diagnosed by renal biopsy. The clinicopathological characteristics of participants were analyzed. Survival analysis was performed using the Kaplan-Meier method. Cox regression analysis and least absolute shrinkage and selection operator (LASSO) regression were conducted to identify risk factors for T2DN progression, and a risk prediction model was constructed for T2DN progression. ROC curves, C-index and calibration curves were used to evaluate the discrimination and calibration of the model. Sensitivity analysis was conducted by redefining MetS using the 2004 Chinese Diabetes Society (CDS) criteria.

**Results:**

The Kaplan-Meier survival curve shows that the cumulative incidence rate of T2DN progression in patients with MetS is significantly higher than in those without MetS (Log-rank test: χ^2^ = 11.76, *P*<0.001). The number of MetS components was an independent risk factor for T2DN progression (HR=2.567, P=0.039; HR=3.392, P<0.001; HR=4.225, P=0.001 for 3,4,5 components respectively). A T2DN progression prediction model by nomogram was constructed, the AUC of ROC curves was 0.794 (95% CI: 0.685-0.908) at 1 year, 0.826 (95% CI: 0.739-0.913) at 2 years, 0.794 (95% CI: 0.694-0.893) at 3 years, and 0.833 (95% CI: 0.735-0.931) at 4 years. the C-index remained above 0.70 for the entire 5-year period. The calibration curves showed a good fit with the reference curves.

**Conclusion:**

MetS is significantly relevant with T2DN progression. Our prediction model helps clinicians to make individualized medical decisions for T2DN patients.

## 1 Introduction

Type 2 diabetes mellitus (T2DM) is a chronic metabolic disease characterized by hyperglycemia, insulin resistance and relative insulin deficiency, which are influenced by both genetic and environmental factors. Its prevalence is continuously rising globally with an estimated prevalence of 12.2% (783.2 million people) in 2045 (1). Due to the increase in the number of diabetes patients, coupled with the improvement in the level of diabetes treatment which has extended the survival time of diabetes patients, the occurrence rate of diabetes complications has significant increased. Diabetic nephropathy (DN) is the most common microvascular complication of diabetes and is also a main cause of end-stage renal disease (ESRD). Due to the lack of obvious clinical symptoms in the early stages of DN, patients are usually already in the later stages of the disease when developing systemic edema, proteinuria, and other manifestations, and rapidly progress to ESRD. The 2012 Kidney Disease: Improving Global Outcomes (KDIGO) Clinical Practice Guideline for the Assessment and Management of Chronic Kidney Disease (CKD) used estimated glomerular filtration rate (eGFR) and urinary albumin-to-creatinine ratio (UACR) as the basis for CKD risk stratification (2). However, subsequent clinical practice has shown that relying solely on eGFR and/or UACR makes it difficult to accurately identify individuals at high risk of progressing to ESRD (3). The latest viewpoint suggests that a comprehensive assessment of patient demographic characteristics, lifestyle factors, comorbidities, etc., should be considered, and accurate predictive models should be used for individualized risk assessment to obtain the risk of progression of kidney function (4). Targeted management strategies based on risk stratification can, to some extent, delay the progression of CKD. As DN is the main cause of CKD, assessing and managing the risk of its progression are crucial for improving CKD prognosis.

Metabolic syndrome (MetS) is a group of interconnected risk factors centered around insulin resistance, triggered by abdominal obesity, including metabolic disorders such as hyperglycemia, hypertension, and hyperlipidemia. Hyperglycemia is an important component of MetS, and it has been shown that about 300 million patients with diabetes will have MetS worldwide by 2025 (5). Additionally, MetS can induce kidney injury through mechanisms such as insulin resistance, abnormal lipid metabolism, oxidative stress, inflammatory response, renin-angiotensin-aldosterone system (RAAS) activation, and endothelial cell dysfunction. The United States Renal Data System (USRDS) suggests that two-thirds of patients who have just entered dialysis have MetS (6). Therefore, in order to verify the correlation between MetS and the occurrence of CKD in T2DM patients, a study involving 5829 Chinese T2DM patients was conducted in Hong Kong. The results indicate that MetS is an independent predictive factor for the occurrence of CKD in T2DM patients (7). Additionally, other studies suggest that an increasing number of MetS components is associated with an elevated risk of developing CKD (8). However, there is currently limited research discussing the relationship between MetS and DN progression, and it remains unclear whether the presence of MetS and the number of MetS components can be used to predict the progression of type 2 diabetic nephropathy (T2DN).

Therefore, our study included the presence of MetS, the number of MetS components, and other potentially related clinical and pathological indicators to identify independent predictive factors of T2DN progression. The aim was to construct an accurate risk prediction model, facilitating clinicians to predict the risk of renal function progression based on baseline data of T2DN patients, thereby developing personalized management and treatment plans.

## 2 Materials and methods

### 2.1 Study population

This study was a retrospective, single center cohort study. We defined diabetes mellitus (DM) according to the criteria proposed by the Diabetes Expert Committee of the World Health Organization (WHO) in 1999, and defined DN according to the criteria proposed by the American Diabetes Association (ADA) in 2023 (9). Patients diagnosed with DN by renal biopsy between June 1, 2015, and June 30, 2023 at the First Affiliated Hospital of Fujian Medical University were included. All patients were aged >18 years. The exclusion criteria for the study were as follows: (1) type 1 diabetes mellitus (T1DM) or other specific types of diabetes mellitus. (2) combined with other primary or secondary glomerular diseases diagnosed by renal biopsy. (3) ESRD at renal biopsy, which defined as baseline eGFR < 15ml/min/1.73m^2^; or have already entered renal replacement therapy; (4) combined with malignant tumor; (5) be pregnant at renal biopsy;(6) baseline data or follow-up data are missing too much. Finally, 130 patients were included. The sample size was calculated based on the Cox regression analysis using PASS 2021 software. Assuming power at 80%, and the confidence level of 95%, the required sample size was 115. In consideration of the dropout rate, 130 patients were deemed appropriate. Included patients were divided into two groups according to the presence or absence of MetS, and followed through December 31, 2023. This study was approved by the ethics committee of the First Affiliated Hospital of Fujian Medical University (MTCA, ECFAH of FMU [2015] 084-2). Written informed consent was obtained from each patient.

### 2.2 Data collection

For each of the included patients, we collected data on as follows: (1) General data, such as age, gender, body mass index (BMI), DM duration, smoking, blood pressure, cardiovascular disease (CVD), cerebrovascular disease, CKD stages and use of angiotensin converting enzyme inhibitors (ACEI)/angiotensin receptor blockers (ARB). (2) Laboratory data: such as fasting blood glucose (FPG), glycosylated hemoglobin, type A1c (HbA1c), total cholesterol (TC), triglycerides (TG); high density lipoprotein cholesterol (HDL-C), low density lipoprotein cholesterol (LDL-C), serum creatinine (Scr), eGFR, UACR, C-reactive protein (CRP), hemoglobin (Hb), blood urea nitrogen (BUN), serum albumin, urinary protein in 24h, serum calcium, serum phosphorus, uric acid, fibrinogen (FIB) and parathyroid hormone (PTH). (3) Pathological feature: All patients underwent renal biopsy and renal histopathological examination. Total number of glomeruli, number of glomeruli with sclerosis, Kimmelstiel-Wilson (K-W) nodules, ratio of interstitial fibrosis and tubular atrophy (IFTA), and interstitial inflammatory cell infiltration area were recorded. Renal interstitial tubules and blood vessels were scored according to the criteria proposed by Renal Pathology Society (RPS) (10), and DN was classified into Grade I-IV according to the pathological Classification of DN proposed by RPS (10). (4) Follow-up data: Follow-up indicators included the last follow-up time, last reassessment of Scr and eGFR, whether endpoint events occur and the time of occurrence of endpoint events.

### 2.3 Outcomes

The starting time point was defined as the date of T2DN diagnosis via renal biopsy, and the patients were followed through December 31, 2023. The primary outcome was occurrence of T2DN progression, defined as (1) a doubling of serum creatinine levels compared to baseline, or ≥50% decline in estimated glomerular filtration rate (eGFR) from baseline; (2) initiation of renal replacement therapy (including kidney transplantation, hemodialysis, peritoneal dialysis) during follow-up.

### 2.4 Some definitions

1. MetS was defined according to the modified criteria of the National Cholesterol Education Program Adult Treatment Panel Third Report (ATP III) (11), MetS status was considered positive if patient had three or more of the following: (a) abdominal obesity (waist circumference >90 cm for men and >80 cm for women), As the participants included in this study did not have routine measurements of waist circumference, BMI was used instead of waist circumference as one of the risk factors according to the diagnostic criteria for MetS proposed by the Chinese Diabetes Society (CDS) in 2004, and BMI >25.0 kg/m^2^ was defined as obesity (12). (b) elevated blood pressure (systolic blood pressure ≥130 mmHg or diastolic blood pressure ≥85 mmHg or use of anti-hypertension medications). (c) increased plasma triglycerides (≥150 mg/dL) or treated dyslipidemia; (d) low fasting HDL-C (men <1.03 mmol/L and women <1.29 mmol/L); (e) elevated fasting glucose (≥6.1 mmol/L) or use of anti-diabetic medication.
2. The 2004 CDS criteria for MetS (12), MetS status was considered positive if patient had three or more of the following: (a) overweight or obesity (BMI>25.0 kg/m^2^). (b) elevated fasting glucose (≥6.1 mmol/L) and/or a 2-hour oral glucose tolerance test (OGTT) result≥7.8 mmol/L, or use of anti-diabetic medication. (c) systolic blood pressure ≥140 mmHg or diastolic blood pressure≥90 mmHg or use of anti-hypertension medications. (d) Fasting plasma triglycerides ≥1.7 mmol/L, and/or low fasting HDL-C (men <0.9 mmol/L and women <1.0 mmol/L).
3. Hypertension was defined according to the criteria of World Health Organization-International Society of Hypertension (WHO/ISH) (13), or the diagnosis have been verified by use of anti-hypertension medications.
4. CVD was defined as a previous diagnosis of heart failure, myocardial infarction, valvular heart disease, percutaneous coronary intervention, or bypass grafting.
5. Cerebrovascular disease was defined as a previous diagnosis of cerebral infarction, cerebral hemorrhage, or stroke.
6. The Scr was measured using an enzymic method though Siemens ADVIA2400. The eGFR was calculated by the CKD-EPI equation (14).

### 2.5 Statistical analysis

R software, version 4.3.2 and SPSS software, version 25.0 was used for statistical analysis. As variables exhibited missingness rates below 30% and met the MCAR assumption (**Supplementary Figure S1**, **Supplementary Table S1**, **Supplementary Figure S2**), multiple imputation was performed using a linear regression model to generate five imputed datasets. Reliability analysis was performed on five new datasets, and the imputed dataset with the largest Cronbach’s coefficient which had the better consistency and stability was selected for subsequent statistical analysis. Normally distributed data are presented as mean ± standard deviation (SD) and compared with Student’s t-test. Non-normally distributed variables are expressed as median (interquartile range [IQR]) and analyzed using the Mann-Whitney U test. Categorical variables were compared by chi-square test. The median follow-up duration was estimated via the reverse Kaplan-Meier method. Kaplan-Meier curves were generated to visualize survival probabilities, with between-group differences in T2DN progression assessed by log-rank test. We determined the relevant factors affecting T2DN progression using the univariate Cox regression analysis. To address multicollinearity and potential overfitting, variables with *P*<0.1 were entered into the LASSO (least absolute shrinkage and selection operator) regression with 10-fold cross-validation. The most predictive variables were determined by 1 standard error criterion (1-SE criteria). Finally, the variables selected through LASSO regression were subsequently included in multivariate Cox regression analysis to construct the final predictive model. The final model was used to construct nomogram for predicting T2DN progression. Receiver operating characteristic (ROC) curves and concordance index (C-index) were used to evaluate the discrimination of the prediction model, while internal validation was performed using Bootstrap resampling. And calibration curves were drawn to assess the calibration of the prediction model. *P*-value<0.05 was considered statistically significant.

## 3 Results

### 3.1 Comparison of clinical characteristics between T2DN patients with/without MetS

Among the 130 T2DN patients included, 79 cases (60.8%) had MetS, while 51 cases (39.2%) did not. Among them, 92 (70.8%) were male and 38 (29.2%) were female. The median age of patients at the time of renal biopsy was 53 (41.75, 61.00) years. The diabetes duration of patients at the time of renal biopsy was 10.00 (4.75, 18.00) years. There were 13 patients (10%), 31 patients (23.8%), 63 patients (48.5%), and 23 patients (17.7%) in CKD stages 1-4, respectively. Among them, the number of patients with MetS in each stage was 6, 18, 36, and 19, accounting for 46.2%, 58.1%, 57.1%, and 82.6% of the total patient population in each respective stage. A total of 70 patients (53.8%) developed renal function progression, including 14 cases (27.5%) in the group without MetS and 56 cases (70.9%) in the group with MetS, which showed that T2DN patients with MetS were more likely to progress than those without MetS (*P*<0.001). Compared with T2DN patients without MetS, T2DN patients with MetS had a higher incidence of obesity, hypertension, CVD, and a more significant increase in BMI and systolic blood pressure (*P*<0.05). The use of ACEI/ARB in T2DN patients without MetS was higher than that in patients with MetS (*P*=0.038), suggesting that ACEI/ARB may have a protective effect in preventing MetS. In terms of laboratory data, compared to the group without MetS, T2DN patients with MetS had higher levels of TC, Scr, BUN, CRP and FIB, and lower levels of HDL-C, eGFR and Hb *(P*<0.05) (**Table 1**).

**Table 1:** Comparison of baseline characteristics in patients with/without MetS.

Variables	All patients (N=130)	With/Without MetS		t/Z/χ^2^ value	*P* value
		Without MetS (N=51)	With MetS (N=79)		
General data					
Age (years)	53.00 (45.75, 61.00)	53.00 (45.00, 61.00)	53.00 (46.00, 61.00)	0.546	0.585
Gender (male, N, %)	92 (70.8%)	41 (80.4%)	51 (64.6%)	3.757	0.053
Smoking (N, %)	47 (36.2%)	19 (37.3%)	28 (35.4%)	0.044	0.834
DM duration (years)	10.00 (4.75, 18.00)	10.00 (3.50, 13.00)	13.00 (5.00, 18.00)	1.776	0.076
BMI (kg/m2)	23.45 (21.78, 24.93)	22.84 (21.44, 24.13)	24.12 (22.35, 25.95)	3.393	0.001^*^
Obesity (N, %)	32 (24.6%)	2 (3.9%)	30 (38.0%)	19.368	<.001^*^
SBP (mmHg)	147.66 ± 24.08	142.18 ± 21.39	151.20 ± 25.17	-2.115	0.036^*^
DBP (mmHg)	82.68 ± 13.98	81.20 ± 13.48	83.63 ± 14.29	-0.970	0.334
Hypertension (N, %)	117 (90.0%)	42 (82.4%)	75 (94.9%)	5.453	0.020^*^
CKD stages (N, %)				10.296	0.036^*^
G1	13 (10.0%)	7 (13.7%)	6 (7.6%)		
G2	31 (23.8%)	13 (25.5%)	18 (22.8%)		
G3a	26 (20.0%)	15 (29.4%)	11 (13.9%)		
G3b	37 (28.5%)	12 (23.5%)	25 (31.6%)		
G4	23 (17.7%)	4 (7.8%)	19 (24.1%)		
Cardiovascular disease (N, %)	48 (36.9%)	13 (25.5%)	35 (44.3%)	4.710	0.030^*^
Cerebrovascular disease (N, %)	12 (9.2%)	3 (5.9%)	9 (11.4%)	1.123	0.289
Use of ACEI/ARB (N, %)	91 (70.0%)	41 (80.4%)	50 (63.3%)	4.316	0.038^*^
Progression (N, %)	70 (53.8%)	14 (27.5%)	56 (70.9%)	23.527	<.001^*^
Laboratory data					
FPG (mmol/L)	7.41 (5.42, 9.74)	6.81 (4.84, 9.88)	7.78 (5.85, 9.60)	1.793	0.073
HbA1c (%)	7.55 (6.40, 9.10)	7.70 (6.40, 10.20)	7.50 (6.40, 8.90)	-0.889	0.374
HbA1c (mmol/mol)	59 (46.4, 76.0)	61 (46.4, 88.0)	58 (46.4, 73.8)	-0.889	0.374
TC (mmol/L)	5.09 (3.81, 6.60)	4.95 (3.86, 6.03)	5.28 (3.71, 7.27)	1.202	0.230
TG (mmol/L)	1.67 (1.09, 2.29)	1.18 (1.02, 1.63)	2.06 (1.60, 3.15)	6.070	<.001^*^
HDL-C (mmol/L)	1.12 (0.90, 1.35)	1.32 (1.13, 1.59)	1.00 (0.77, 1.21)	-5.928	<.001^*^
LDL-C (mmol/L)	3.20 (2.21, 4.40)	3.16 (2.13, 4.14)	3.23 (2.26, 4.90)	0.722	0.470
Scr at first (μmol/L)	131.50 (99.08, 168.08)	123.30 (96.60, 157.80)	142.80 (103.00, 206.00)	2.053	0.040^*^
eGFR at first (ml/min/1.73m^2^)	46.98 (37.43, 70.75)	53.40 (41.50, 76.10)	43.30 (31.10, 65.42)	-2.794	0.005^*^
UACR (mg/g)	3175.93 (1727.79, 5626.66)	2695.84 (1414.35, 4738.37)	3606.14 (2033.61, 6202.88)	1.671	0.095
CRP (mg/L)	2.90 (1.55, 6.33)	1.97 (1.08, 5.00)	3.59 (2.14, 7.20)	3.343	0.001^*^
Hb (g/L)	104.50 (93.00, 120.50)	109.00 (102.00, 126.00)	100.00 (91.00, 116.00)	-2.344	0.019^*^
BUN (mmol/L)	9.56 (6.80, 13.18)	7.80 (6.22, 12.68)	10.16 (7.60, 15.06)	2.065	0.039^*^
Serum albumin (g/L)	30.80 ± 6.74	31.45 ± 6.25	30.38 ± 7.04	0.876	0.382
Urinary protein in 24h (g/24h)	4.20 (2.41, 7.45)	3.82 (1.87, 7.35)	4.61 (2.44, 7.59)	0.827	0.408
Calcium (mmol/L)	2.07 (1.98, 2.18)	2.12 (2.01, 2.23)	2.05 (1.96, 2.16)	-1.910	0.056
Phosphorus (mmol/L)	1.27 (1.12, 1.44)	1.28 (1.12, 1.44)	1.26 (1.12, 1.44)	-0.250	0.802
FIB (g/L)	4.64 (3.86, 5.60)	4.25 (3.40, 5.22)	4.86 (4.11, 6.30)	2.716	0.007^*^
PTH (pmol/L)	4.54 (2.73, 6.96)	3.53 (2.63, 6.39)	5.03 (2.91, 8.41)	1.483	0.138
Uric acid (μmol/L)	381.85 (324.60, 434.25)	360.00 (325.00, 422.20)	388.00 (323.40, 445.40)	1.507	0.132
Scr at end point (μmol/L)	274.50 (130.00, 542.43)	143.00 (96.00, 351.80)	337.00 (202.00, 652.00)	4.022	<.001^*^
eGFR at end point (ml/min/1.73m^2^)	21.50 (8.10, 51.60)	40.40 (15.20, 72.70)	13.20 (7.20, 28.30)	-4.358	<.001^*^
Pathological feature					
Glomerulosclerosis rate (%)	38.05 (13.30, 66.70)	33.30 (11.40, 60.00)	40.00 (14.30, 69.20)	1.095	0.273
K-W nodules (N, %)	85 (65.4%)	27 (52.9%)	58 (73.4%)	5.741	0.017^*^
Pathological Classification (N, %)				11.397	0.022^*^
I	5 (3.8%)	5 (9.8%)	0 (0.0%)		
IIa	21 (16.2%)	11 (21.6%)	10 (12.7%)		
IIb	19 (14.6%)	8 (15.7%)	11 (13.9%)		
III	58 (44.6%)	19 (37.3%)	39 (49.4%)		
IV	27 (20.8%)	8 (15.7%)	19 (24.1%)		
IFTA scores(N, %)				2.812	0.245
0/1	40 (30.8%)	20 (39.2%)	20 (25.3%)		
2	52 (40.0%)	18 (35.3%)	34 (43.0%)		
3	38 (29.2%)	13 (25.5%)	25 (31.6%)		
Renal interstitial inflammation (N, %)				0.001	0.971
0/1	125 (96.2%)	49 (96.1%)	76 (96.2%)		
2	5 (3.8%)	2 (3.9%)	3 (3.8%)		
Vascular scores (N, %)				12.987	0.005^*^
0	5 (3.8%)	4 (7.8%)	1 (1.3%)		
1	8 (6.2%)	7 (13.7%)	1 (1.3%)		
2	113 (86.9%)	38 (74.5%)	75 (94.9%)		
3	4 (3.1%)	2 (3.9%)	2 (2.5%)		

Data are expressed as means eastandard deviation or medians (interquartile range) or count (%).

^*^ *P* value<0.05.

MetS, metabolic syndrome; DM, diabetes mellitus; BMI, body mass index; SBP, systolic blood pressure; DBP, diastolic blood pressure; CKD, chronic kidney disease; ACEI, angiotensin converting enzyme inhibitors; ARB, angiotensin receptor blockers; FPG, fasting blood glucose; HbA1c, glycosylated hemoglobin, type A1c; TC, total cholesterol; TG, triglycerides; HDL-C, high density lipoprotein cholesterol; LDL-C, low density lipoprotein cholesterol; Scr, serum creatinine; eGFR, estimated glomerular filtration rate; UACR, urinary albumin to-creatinine ratio; CRP, C-reactive protein; Hb: hemoglobin; BUN, blood urea nitrogen; FIB, fibrinogen; PTH, parathyroid hormone; IFTA, interstitial fibrosis and tubular atrophy.

### 3.2 Comparison of pathological characteristics between T2DN patients with/without MetS

The pathological grade of RPS in T2DN patients was mainly Grade III, accounting for 58 cases (44.6%). Additional, as the pathological grade of RPS increased, the proportion of patients with MetS also increased (*P*=0.022). Compared to patients without MetS, those with MetS had a higher proportion of K-W nodules and more severe renal vascular lesions (*P* < 0.05) (**Table 1**).

### 3.3 Survival curve by Kaplan-Meier analysis

The median follow-up time evaluated by reverse Kaplan-Meier analysis was 35.60 (24.20, 47.00) months. 70 patients entered endpoint events in total, including 14 patients (27.5%) without MetS and 56 patients (70.9%) with MetS, and the difference was statistically significant (P<0.001). The median non-progression time evaluated by Kaplan-Meier analysis was 29.67 (24.71, 34.63) months (**Table 2**).

**Table 2:** Influence of MetS on renal terminal events.

Events	Yes/No	Total (N=130)	With/Without MetS		*P* value
			Without MetS (N=51)	With MetS (N=79)	
Progression (N, %)	Yes	70 (53.8%)	14 (27.5%)	56 (70.9%)	<.001^*^
	No	60 (46.2%)	37 (72.5%)	23 (29.1%)	
Median non-progression time (months)		29.67 (24.71, 34.63)	45.40 (32.42, 58.38)	22.17 (15.41, 28.93)	–
Median follow-up time (months)		35.60 (24.20, 47.00)	20.37 (11.15, 29.59)	53.73 (37.14, 70.32)	–

MetS, metabolic syndrome.

^*:^P value<0.05.

**Figure 1:**
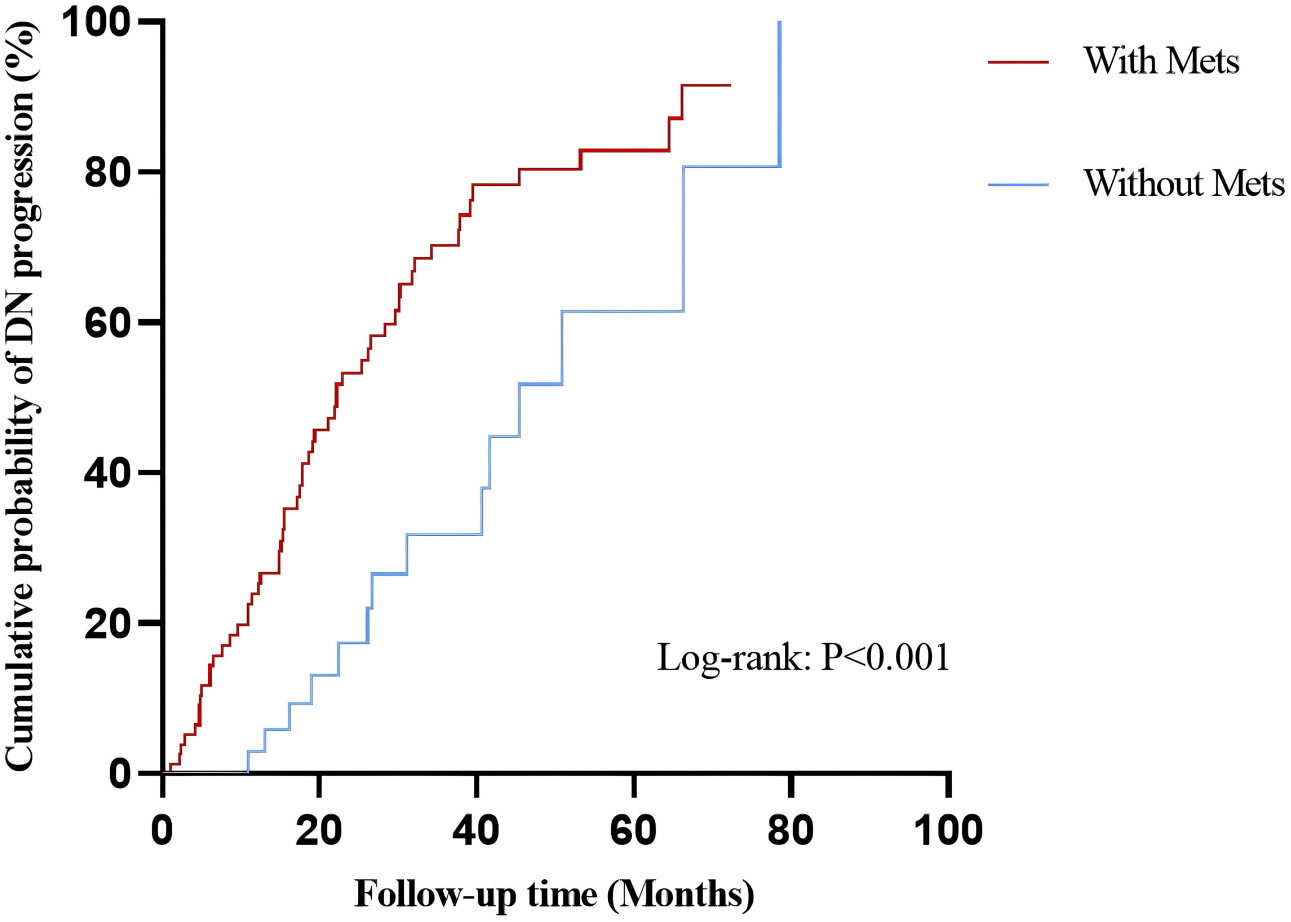
Comparison of cumulative probability of DN progression in patients with/without MetS. DN, diabetic nephropathy; MetS, metabolic syndrome.

The survival curves of patients between MetS subgroups were shown in **Figure 1**. The Kaplan-Meier analysis revealed that patients with MetS had a higher possibility of T2DN progression when compared with those without MetS (Log-rank test: χ^2^ = 11.76, *P*<0.001).

### 3.4 Risk prediction model for T2DN patients

#### 3.4.1 Variables selection for risk prediction model

**Figure 2:**
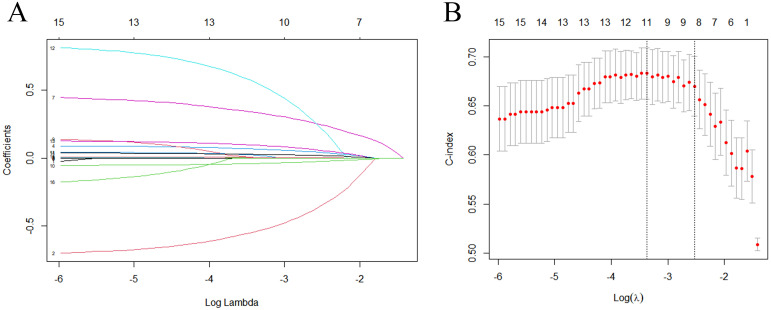
LASSO regression analysis of variables selected from univariate regression analysis. **(A)** LASSO coefficient profiles of the variables via penalized maximum likelihood with L1-norm regularization. **(B)** LASSO regression model was performed by 10-fold cross-validation method. Use the minimum standard (left dotted line) and 1-standard error (SE) criteria (right dotted line) to draw a vertical dashed line at the optimal value. The optimal lambda produced 9 nonzero coefficients (right dotted line, λ = 0.080).

**Table 3** showed the univariate analysis of all the clinical and pathological features. We found that DM duration, obesity, FPG, TG, Scr, eGFR, BUN, serum albumin, urinary protein in 24h, FIB, use of ACEI/ARB, anemia, hypocalcemia, MetS, number of MetS components, glomerulosclerosis rate, IFTA scores were risk factors for T2DN progression. Variables with *P*<0.1 were entered into the LASSO regression analysis with 10-fold cross-validation (**Figures 2A, B**). We used the 1-standard error (SE) criteria (right dotted line in **Figure 2B**) to draw a vertical dashed line at the optimal value. The optimal lambda (λ = 0.080) produced 9 nonzero coefficients. Consequently, the 9 variables with non-zero coefficients were included in multivariate Cox regression analysis. The results showed that FPG, 24-hour urinary protein, FIB, hyperphosphatemia, use of ACEI/ARB, and number of MetS components were independent risk factors for T2DN progression (*P* < 0.05) (**Table 4**).

**Table 3:** Univariate Cox regression analysis of factors affecting the development of DN.

Variables	Univariate regression analysis	
	HR (95%CI)	*P* value
Basic demographic variables		
Gender	0.785 (0.474-1.303)	0.349
Age	0.994 (0.975-1.013)	0.535
Smoking	0.913 (0.558-1.495)	0.717
DM duration	1.030 (1.001-1.060)	0.045^*^
BMI	1.034 (0.940-1.137)	0.492
Obesity	1.721 (1.039-2.851)	0.035^*^
Laboratory data		
HbA1c	1.081 (0.969-1.207)	0.163
FPG	1.112 (1.042-1.186)	0.001^*^
TC	1.074 (0.994-1.161)	0.070
TG	1.140 (1.014-1.282)	0.028^*^
HDL-C	0.793 (0.466-1.350)	0.393
LDL-C	1.066 (0.960-1.183)	0.234
Scr	1.005 (1.002-1.008)	0.001^*^
eGFR	0.986 (0.975-0.996)	0.008^*^
BUN	1.089 (1.047-1.133)	<.001^*^
Serum albumin	0.937 (0.901-0.974)	0.001^*^
urinary protein in 24h	1.12 (1.049-1.195)	0.001^*^
FIB	1.189 (1.083-1.305)	<.001^*^
Clinical characteristics		
Use of ACEI/ARB	0.490 (0.300-0.802)	0.005^*^
Hypertension	1.210 (0.485-3.017)	0.683
Cardiovascular disease	1.146 (0.708-1.855)	0.580
Cerebrovascular disease	0.645 (0.259-1.605)	0.346
Anemia	2.372 (1.131-4.975)	0.022^*^
Hyperuricemia	1.023 (0.634-1.652)	0.925
Hypocalcemia	1.754 (1.080-2.846)	0.023^*^
Hyperphosphatemia	1.609 (0.928-2.790)	0.090
MetS	2.756 (1.506-5.044)	0.001^*^
Number of MetS components (compared with 1 or 2 components including DM)		
3 components	1.563 (0.683-3.577)	0.290
4 components	3.336 (1.753-6.350)	<0.001^*^
5 components	3.276 (1.508-7.115)	0.003^*^
Pathological feature		
Glomerulosclerosis rate	1.009 (1.001-1.017)	0.022^*^
K-W nodules	1.178 (0.698-1.988)	0.539
IFTA scores (compared with 0 or 1 point)		
2 points	1.956 (1.049-3.647)	0.035^*^
3 points	1.962 (1.027-3.746)	0.041^*^
Renal interstitial inflammation scores (compared with 0 or 1 point)	0.375 (0.091-1.537)	0.173
Vascular scores (compared with 0 point)		
1 point	1.030 (0.171-6.191)	0.975
2 points	1.648 (0.402-6.760)	0.488
3 points	0.499 (0.068-3.661)	0.494

^*^ *P* value<0.05.

DN, diabetic nephropathy; DM, diabetes mellitus; BMI, body mass index; HbA1c, glycosylated hemoglobin, type A1c; FPG, fasting blood glucose; TC, total cholesterol; TG, triglycerides; HDL-C, high density lipoprotein cholesterol; LDL-C, low density lipoprotein cholesterol; Scr, serum creatinine; eGFR, estimated glomerular filtration rate; BUN, blood urea nitrogen; FIB, fibrinogen; ACEI, angiotensin converting enzyme inhibitors; ARB, angiotensin receptor blockers; MetS, metabolic syndrome; IFTA, interstitial fibrosis and tubular atrophy.

**Table 4:** LASSO regression analysis and multivariate Cox regression analysis of factors affecting the development of DN.

Variables	LASSO regression analysis λ (1-SE) = 0.080	Multivariate regression analysis	
	Coefficients	HR (95%CI)	*P* value
Use of ACEI/ARB	-0.358	0.581 (0.346-0.975)	0.040^*^
DM duration	0.001	–	–
FPG	0.043	1.094 (1.014-1.180)	0.020^*^
Number of MetS components (compared with 1 or 2 components including DM)	0.250		
3 components		2.567 (1.048-6.288)	0.039^*^
4 components		3.392 (1.737-6.622)	<0.001^*^
5 components		4.225 (1.815, 9.837)	0.001^*^
Serum albumin	-0.024	–	–
Hyperphosphatemia	0.232	2.348 (1.260-4.377)	0.007^*^
FIB	0.059	1.153 (1.015-1.310)	0.029^*^
Urinary protein in 24h	0.020	1.086 (1.009-1.168)	0.027^*^
Glomerulosclerosis rate	0.003	–	–

^*^ *P* value<0.05.

LASSO, Least absolute shrinkage and selection operator; DN, diabetic nephropathy; DM, diabetes mellitus; FPG, fasting blood glucose; FIB, fibrinogen; ACEI, angiotensin converting enzyme inhibitors; ARB, angiotensin receptor blockers; MetS, metabolic syndrome; HR, Hazard Ratio; SE, standard error.

#### 3.4.2 Construction of the prediction model

**Figure 3:**
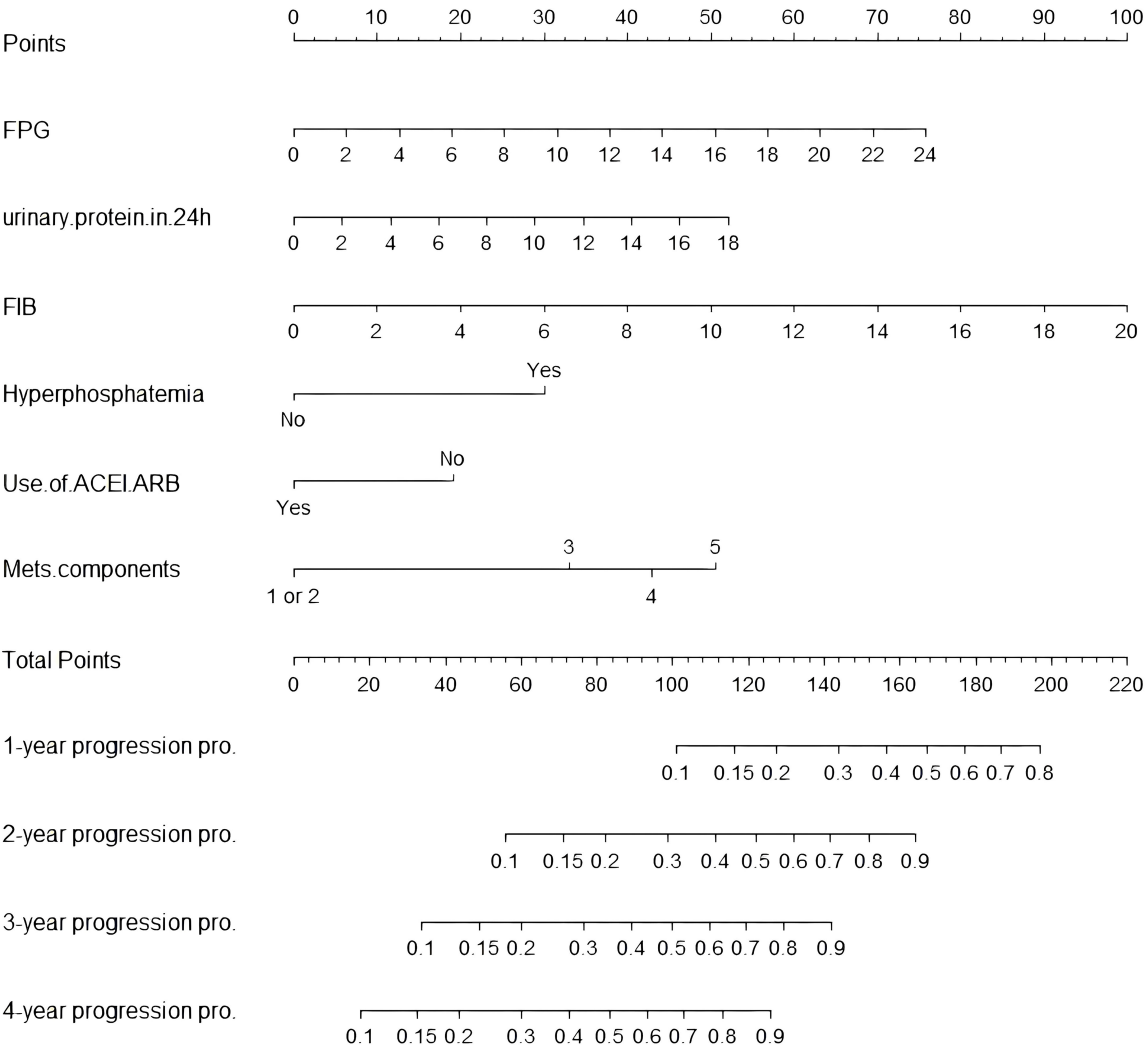
Nomogram for predicting probability of renal function progression in T2DN patients. FPG, fasting blood glucose; FIB, fibrinogen; ACEI, angiotensin converting enzyme inhibitors; ARB, angiotensin receptor blockers; MetS, metabolic syndrome.

The independent predictors selected by multivariate analysis were used to construct a T2DN progression risk prediction model by nomogram (**Figure 3**). To utilize the nomogram, locate each predictor value on its respective axis and draw vertical lines to the Points scale. Sum the assigned points to obtain the Total Points. Locate this sum on the Total Points axis and project vertically to the Progression Probability axis, estimating 1- to 4-year risks of T2DN progression. For example, consider a T2DN patient with these baseline characteristics: FPG 8 mmol/L, 24h urinary protein 6 g/L, FIB 4 g/L, hyperphosphatemia, ACEI/ARBs therapy, and 4 MetS components. The corresponding points are: FPG (25), 24h urinary protein (17.5), FIB (20), hyperphosphatemia (30), ACEI/ARBs therapy (0), and MetS components (42.5). The total points sum to 135, corresponding to projected progression risks of 25% at 1 year, 63% at 2 years, 84% at 3 years, and >90% at 4 years.

#### 3.4.3 Discrimination and calibration of the prediction model

**Figure 4:**
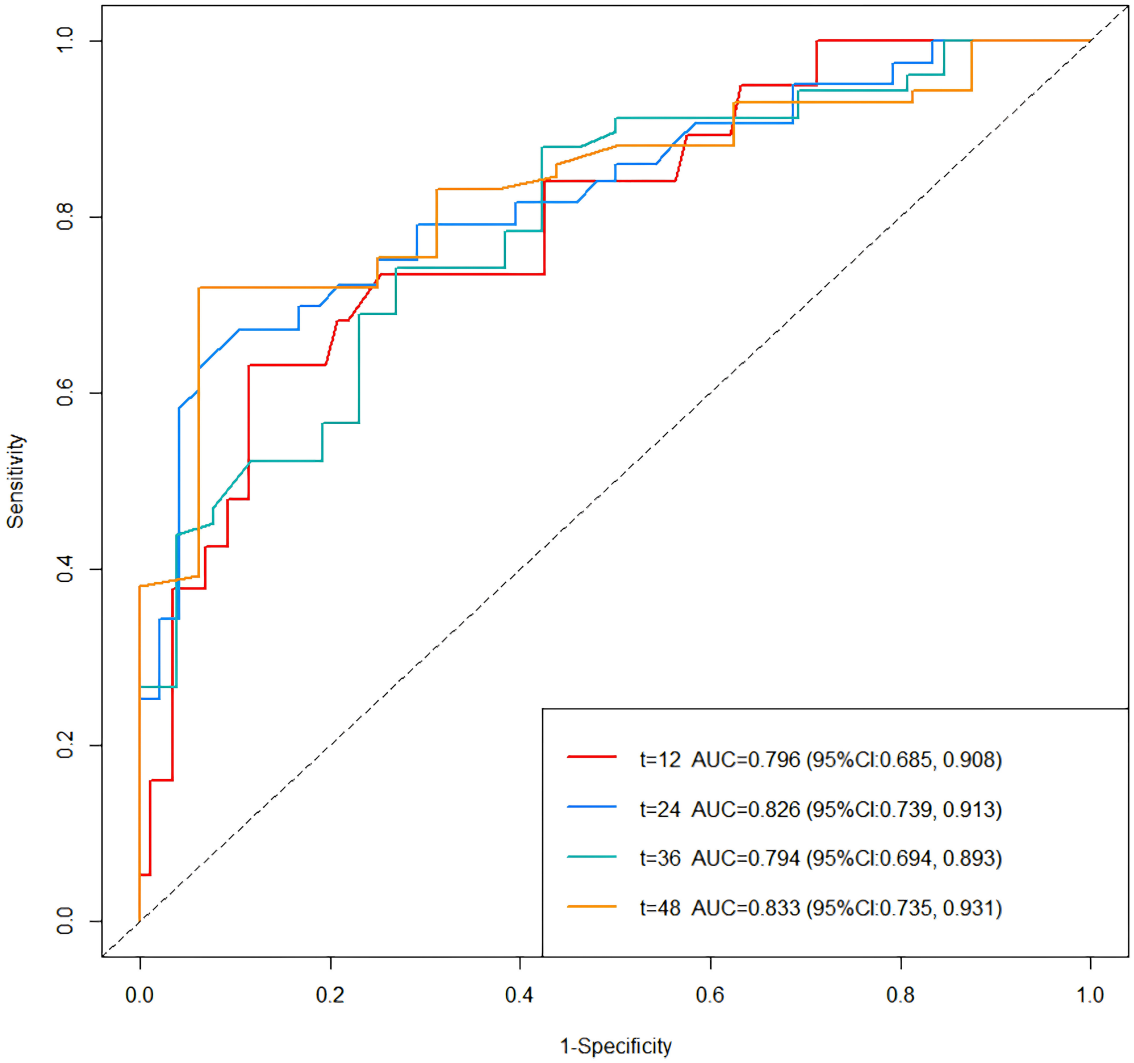
Time-dependent ROC curves for predictive model.

**Figure 5:**
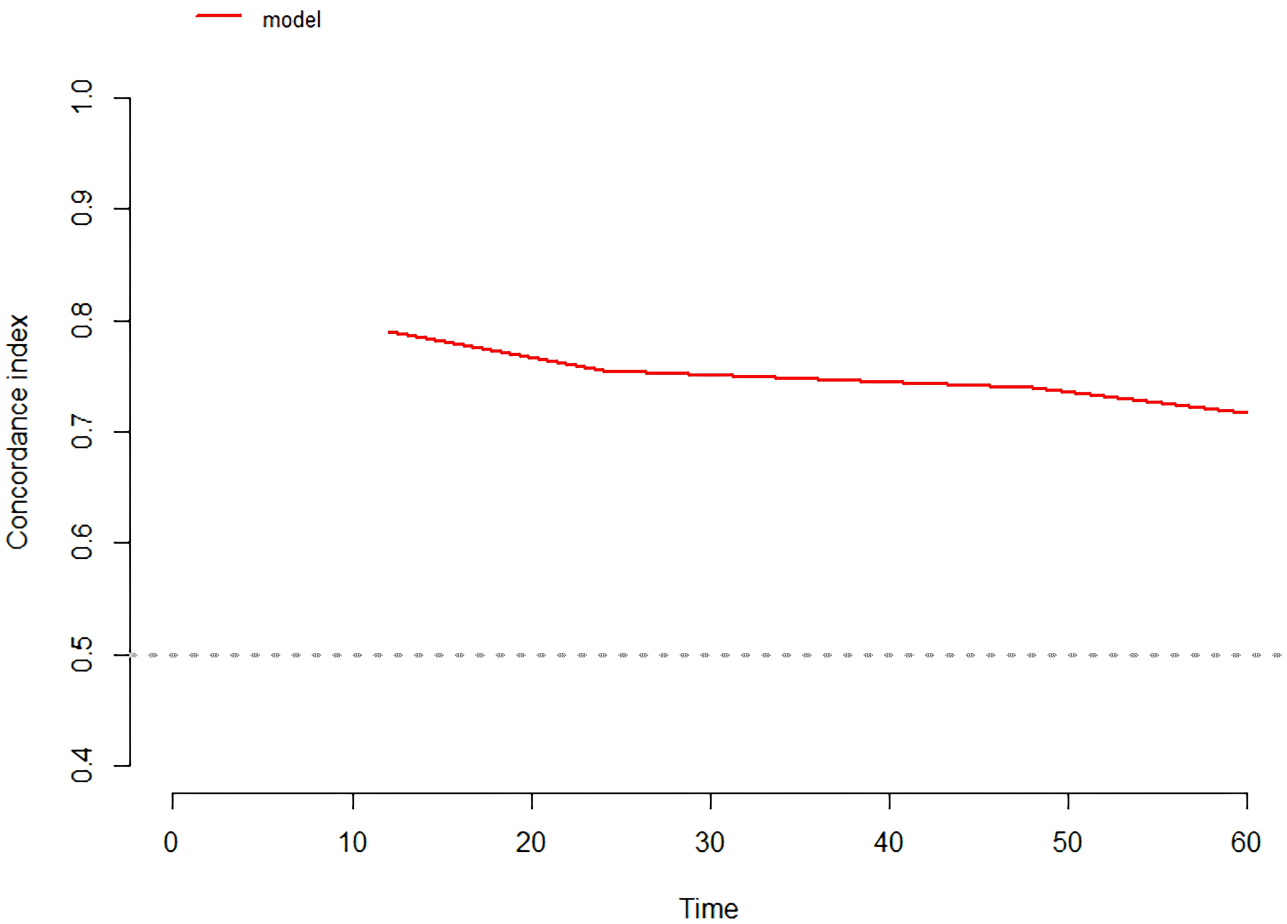
C-index of the prediction model.

**Figure 6:**
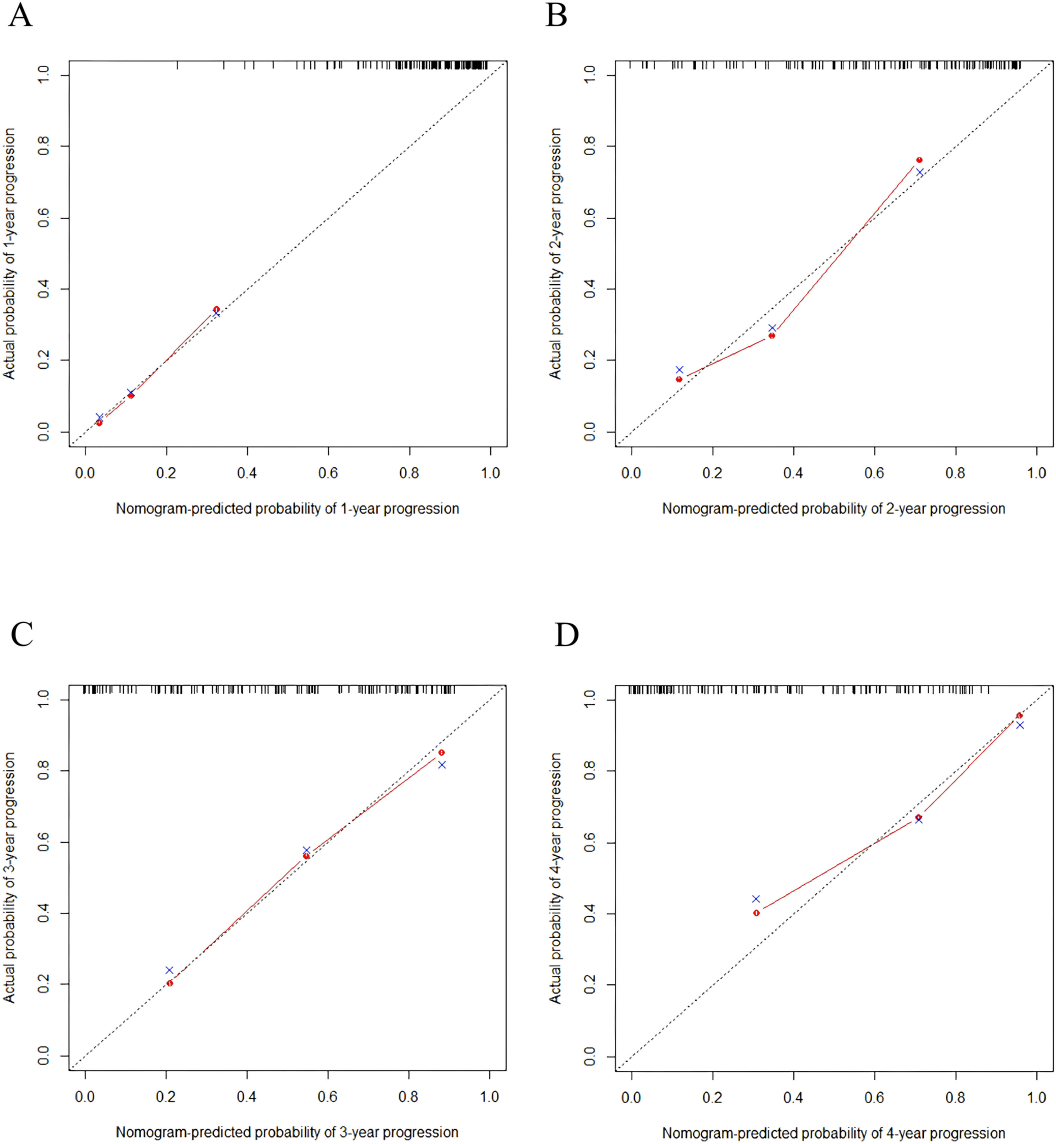
**(A)** 1 year/ **(B)** 2 years/ **(C)** 3 years/ **(D)** 4 years calibration curves for predictive model.

Time-dependent ROC curves were plotted, revealing an AUC of 0.796 (95% CI: 0.685-0.908) at 1 year, 0.826 (95% CI: 0.739-0.913) at 2 years, 0.794 (95% CI: 0.694-0.893) at 3 years, and 0.833 (95% CI: 0.735-0.931) at 3 years (**Figure 4**). C-index values at different time points were calculated and cross-validated using Bootstrap resampling. The results were graphically represented, showing that the C-index of the model remained above 0.7 for the entire 5-year period (**Figure 5**). Both the ROC curves and C-index results indicated that the model has good discriminatory ability. The calibration curves for 1 year, 2 years, 3 years and 4 years showed a good fit with the reference curves, suggesting that the model is well-calibrated (**Figure 6**).

#### 3.4.4 Sensitivity analysis

A sensitivity analysis was conducted to assess the robustness of predictive model by redefining MetS using the 2004 CDS diagnostic criteria. The univariate analysis showed that MetS and the number of MetS components remained the risk factors for T2DN progression (**Supplementary Table S3**). Variables selected from univariate regression analysis were then subjected to LASSO regression. The 10 variables with non-zero coefficients were included in multivariate Cox regression analysis. The results also showed that FPG, 24-hour urinary protein, FIB, hyperphosphatemia, use of ACEI/ARB, and number of MetS components were independent risk factors for T2DN progression (*P* < 0.05) (**Supplementary Table S4**). Following the reclassification of MetS, the reconstructed model exhibited comparable discriminative performance to the primary model (**Supplementary Figure S5**). AUC comparison at 1 year: 0.796 vs 0.789, ΔAUC=0.007, *P*=0.976 by DeLong’s test; 2 years: 0.826 vs 0.797, ΔAUC=0.029, *P*=0.180 by DeLong’s test; 3 years: 0.794 vs 0.796, ΔAUC=0.002, *P*=0.388 by DeLong’s test; 4 years: 0.833 vs 0.844, ΔAUC=0.011, *P*=0.913 by DeLong’s test. Redefining MetS per CDS-2004 criteria did not alter model discriminability, supporting the stability of our model.

## 4 Discussion

To our knowledge, this is the first study assessing MetS as one of the factors to construct the risk prediction model for T2DN progression. Our study suggested that MetS was significantly relevant with T2DN progression, and the number of MetS components was one of the independent risk factors for T2DN progression. We also found that FPG, 24-hour urinary protein, FIB, hyperphosphatemia, use of ACEI/ARB were also independent risk factors for T2DN progression, and we used these independent risk factors to construct a risk prediction model that can be used to predict the probability of T2DN progression.

The prevalence of MetS in 130 adult T2DN patients included in this study was 60.8%. The result showed that the use of ACEI/ARBs was higher in T2DN patients without MetS than in patients with MetS. Previous studies demonstrated that ARBs activate peroxisome proliferator-activated receptor gamma (PPARγ) (15, 16), thereby improving insulin sensitivity and potentially mitigating MetS development. A prospective cohort study in hypertension patients revealed that ARBs appeared to improve the lipid parameters and exert beneficial effects on lipid metabolism and MetS (17). It can be reasonably inferred that use of ACEI/ARBs may have a protective effect in preventing MetS. As CKD progressed from G1 to G5, the prevalence of MetS also increased. A study from the National Kidney Foundation’s Kidney Early Evaluation Program (KEEP) indicated that, compared to African Americans, white individuals had a higher prevalence of MetS, with a significant increase in prevalence of CKD G3-G5. Conversely, African Americans had a lower prevalence of MetS, with a higher prevalence of CKD G1-G2 (18), which is consistent with the results of our study, suggesting a potential association between MetS and adverse outcomes in T2DN. Furthermore, the pathological grades of T2DN patients with MetS were mainly Grade III and Grade IV. In recent years, multiple studies have shown that the incidence of DN progressing to ESRD increases as the pathological grading of DN increases (19, 20). It can be reasonably inferred that T2DN patients with concurrent MetS have a higher risk of adverse renal outcomes. Univariate Cox regression analysis suggested that the components of MetS, such as obesity, FPG, and TG, were all correlated with the prognosis of T2DN. However, after adjusting for all predictive factors selected from the univariate Cox regression analysis, obesity and TG were not independent predictors of T2DN progression. Although FPG could be considered an independent predictor, its HR value was lower than that for the number of MetS components, indicating that MetS was a more reliable independent predictor of T2DN prognosis compared to obesity, FPG, and TG.

MetS have previously been shown to be strongly associated with the development of CKD. A cross-sectional study involving 4933 African American individuals showed that MetS is an independent risk factor for developing CKD, and the study also evaluated the predictive ability of various combinations of MetS components for CKD occurrence. The results indicated that combinations including FPG exhibited strong predictive abilities, which could be corroborated with the finding of this study that FPG is an independent predictor of T2DN progression (8). A study conducted among urban populations in China also indicated that the presence of MetS and the number of MetS components were independently associated with the occurrence of CKD. Among the components, FPG was identified as the primary cause of declining kidney function (21). There were also some studies investigated the relationship between MetS and the progression of CKD. A cohort study involving 3108 Chinese community-based residents indicated a close association between MetS and rapid decline in eGFR (eGFR decline >3 ml/min/1.73m2) (22). T2DM is not only an important cause of CKD, but also a key component of MetS. However, few studies have discussed the relationship between MetS and T2DN progression. A retrospective study by Edgar et al. suggested that treating MetS can delay the progression of DN (23). Our study further confirmed that the presence of MetS and the increased number of MetS components can independently predict the progression of T2DN. Based on the results of our study, we believe that prevention and early treatment of MetS is essential to control the T2DN progression. In addition to strict management of blood glucose levels, metabolism-related diseases such as obesity, hypertension, and hyperlipidemia should also be given attention in order to reduce the number of MetS components.

Microalbuminuria is an important feature in the early stage of DN, and 24-hour urinary protein quantification is the gold standard for assessing proteinuria levels in DN patients. Our study showed that the increase of urinary protein was an independent risk factor for T2DN progression, which was consistent with the conclusions of several previous studies (24–26). And a large number of randomized controlled trials have suggested that urinary protein levels can be reduced by the use of drugs with renoprotective effects or by reducing protein intake and low-sodium diet, thereby improving the cardiorenal prognosis of T2DN patients (27).

The renal protective effect of ACEI/ARB drugs is mainly to reduce the proteinuria by dilating the afferent arteriole of the glomerulus, reducing the glomerular effective filtration pressure, and improving the permeability of the glomerular filtration membrane, as well as delaying glomerular fibrosis by inhibiting glomerular mesangial cell proliferation. Our study suggested that the use of ACEI/ARB was an independent protective factor which can delay the T2DN progression. The use of ACEI/ARB has become globally recognized as an important treatment for DN. 2022 KDIGO Clinical Practice Guideline for the Management of Diabetes in Chronic Kidney Disease pointed out that it was also reasonable for DN patients with normal blood pressure, but presence of proteinuria to select ACEI/ARB for treatment (28). A meta-analysis suggested that ARB were superior to ACEI in reducing renal and cardiovascular events in DN patients (29). This study did not discuss the differences between ACEI and ARB in improving the prognosis of T2DN, and further study could be conducted on this aspect in the future.

The excretion of phosphate is almost entirely dependent on the excretory function of the kidneys, and the progressive decline in renal function often accompanies the development of hyperphosphatemia. In this study, we found that hyperphosphatemia was an independent risk factor for T2DN progression. Xiang et al. conducted a follow-up study on 591 patients with DN and 957 patients with IgA nephropathy to evaluate the impact of hyperphosphatemia on the progression of CKD due to different causes. The results suggested that hyperphosphatemia was an independent risk factor for DN progression, while its relationship with the progression of IgA nephropathy was not statistically significant (30). A retrospective cohort study suggested that the risk of ESRD increased with each 0.5 mg/dl increase when serum phosphorus levels were greater than 3 mg/dl (31). In patients with renal insufficiency who develop hyperphosphatemia, there is often accompanying hypocalcemia, elevated fibroblast growth factor-23 (FGF-23) levels, and decreased active vitamin D levels. Prolonged stimulation of the parathyroid glands by factors such as hypocalcemia and hyperphosphatemia can lead to excessive secretion of PTH, resulting in secondary hyperparathyroidism. To determine whether hyperphosphatemia itself or secondary hyperparathyroidism accelerates the progression of renal function, a retrospective study involving 2445 CKD patients found that both hyperphosphatemia and the presence of secondary hyperparathyroidism were independently associated with the progression of CKD after adjustment for multiple factors (32).

Fibrinogen is considered to be a ligand for intercellular cell adhesion molecule-1 (ICAM-1) on the surface of endothelial cells and Toll-like receptors 4 (TLR4) on podocytes and inflammatory cells. The binding of fibrinogen to ICAM-1 and TLR4 can regulate endothelial permeability and induce podocyte damage and inflammation (33–35), thereby causing kidney injury. Our study suggested that serum fibrinogen was an independent risk factor for T2DN progression. Currently, there is still limited research on the relationship between fibrinogen and the progression of DN. Zhang et al. stratified serum fibrinogen into quartiles to explore its impact on the prognosis of T2DN (36). The results suggested that baseline serum fibrinogen levels were negatively correlated with eGFR levels, positively correlated with proteinuria and serum cholesterol levels. Additionally, serum fibrinogen was identified as an independent risk factor for the progression of T2DN to ESRD, which was consistent with the findings of this study.

Currently, clinical indicators to achieve early individualized warnings for the progression of T2DN patients remains a challenge. Our study further constructed a risk prediction model including six clinical predictors for T2DN progression, and nomogram was used to quantify the model to help nephrologists more accurately assess the risk of adverse renal outcomes in each patient and make targeted clinical decisions. In order to make the model easier to use and integrate the model into clinical practice, we will develop a dynamic online nomogram in the future. Using the dynamic online nomogram based on the prediction model, patients and nephrologists could conveniently obtain individualized T2DN progression probability via a website. Once high-risk patients are identified by the model, the referral to nephrology specialists is necessary, and some interventions should be performed, such as strict control of weight, blood glucose, blood pressure, TG, HDL, urinary protein and serum phosphorus level. The early identification of high-risk T2DN patients and the accurate interventions might reduce patients’ exposure to risk factors, and help prevent T2DN progression.

Nevertheless, it should be noted that some limitations exist in our study. First of all, it was a single center, retrospective study with limited sample size. The patient population from a single center typically represents a specific geographic region and the diagnostic and treatment practices of a particular healthcare institution. Consequently, it might not fully represent the target population for which the predictive model is intended to be applied, leading to selection bias in model development. During retrospective data collection, information bias might arise in building the predictive model due to participants’ recall bias or omissions of information. And we had not yet obtained suitable, independent, and sufficiently large external cohort data to perform external validation of the model. The generalizability of the findings to broader populations would be limited. Once sufficient independent cohort data is obtained, we will rigorously conduct an external validation to make the findings more compelling. It must be stressed that the model is not ready for direct application in guiding clinical decision-making until external validation has been completed. Second, we use multiple imputation method to deal with missing data, which may have some deviation from the true data. Third, the participants included in this study did not have routine measurements of waist circumference, BMI was used instead of waist circumference as the assessment criterion for central obesity. Previous studies have found that the Chinese population generally has a higher percentage of body fat and a greater propensity to abdominal obesity compared with those of western counterparts under the same BMI (37), and individuals having normal BMI with MetS are not rare in the Chinese population (38). Based on the ATPIII definition, the substitution of BMI for waist circumference may lead to underdetection of central obesity, resulting in misclassification of some MetS patients into the non-MetS group. The study results may exhibit certain discrepancies when compared with cohorts using waist circumference-based MetS diagnosis. Following the establishment of prospective external validation cohorts, we will conduct sensitivity analyses to assess variations in group classification and model performance under BMI-based versus waist circumference-based MetS diagnostic criteria. Finally, various new drugs for treating T2DN have emerged recently, all of which have been shown to have good renal protective effects, such as sodium-dependent glucose transporters 2 (SGLT-2) inhibitors, non-steroidal selective mineralocorticoid receptor antagonists, etc. However, our study has not yet explored the effects of these new drugs on T2DN prognosis. We commit to expanding the cohort and further investigating the effects of emerging therapies on DN progression after collecting data on new therapeutic regimens.

## 5 Conclusion

MetS was significantly relevant with T2DN progression, and the number of MetS components was one of the independent risk factors for T2DN progression. We used the independent risk factors selected by multivariate analysis, including FPG, 24-hour urinary protein, FIB, hyperphosphatemia, use of ACEI/ARB and the number of MetS components, to construct a risk prediction model to help clinicians to make individualized medical decisions for T2DN patients. Further large-scale prospective studies are still required to validate our prediction model and enhance the generalizability of our findings.

## Data Availability

The raw data supporting the conclusions of this article will be made available by the authors, without undue reservation.
