# Supplementary material for: Risk prediction model for progression of type 2 diabetic nephropathy with and without metabolic syndrome: a retrospective cohort study
Source: Front Endocrinol (Lausanne). 2025 Jul 30;16:1592180. doi: 10.3389/fendo.2025.1592180 (PMC12343258; doi:10.3389/fendo.2025.1592180)
Supplement: Supplementary file 1 [file DataSheet1.docx]

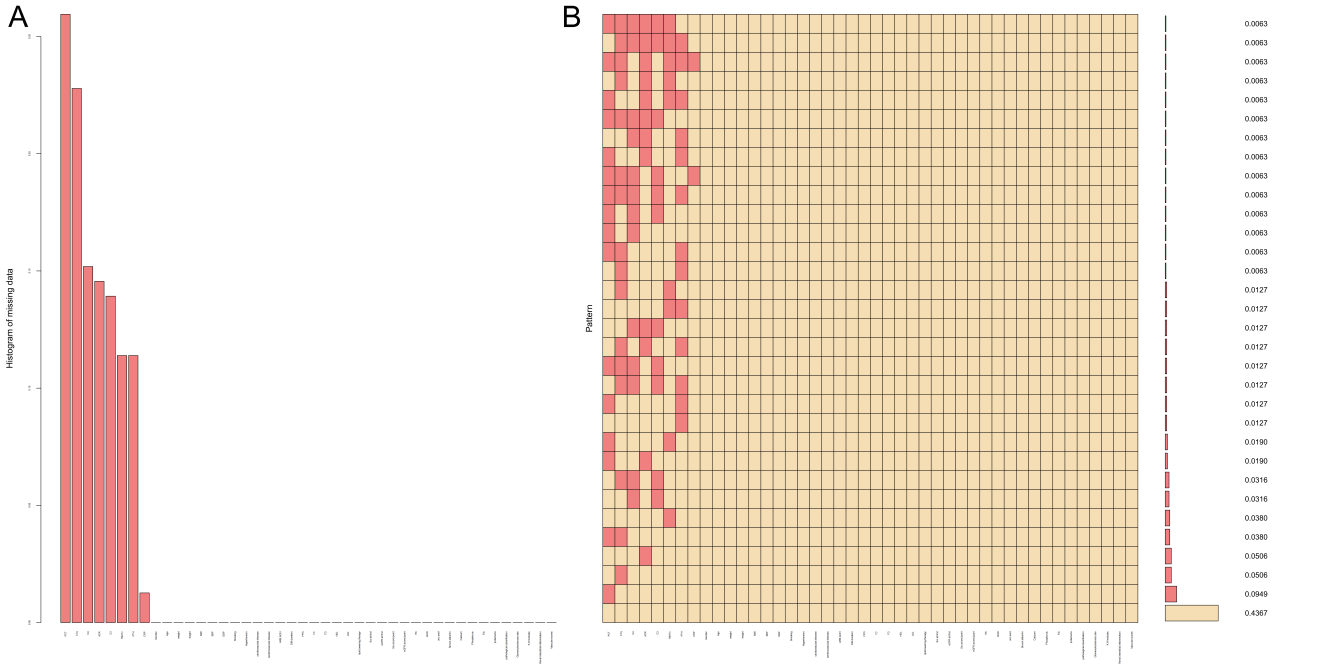


**Figure S1.** The extent and pattern of missing data.


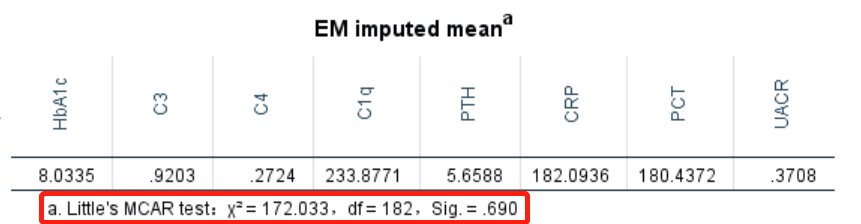


**Figure S2.** Little’s MCAR test for missing data.


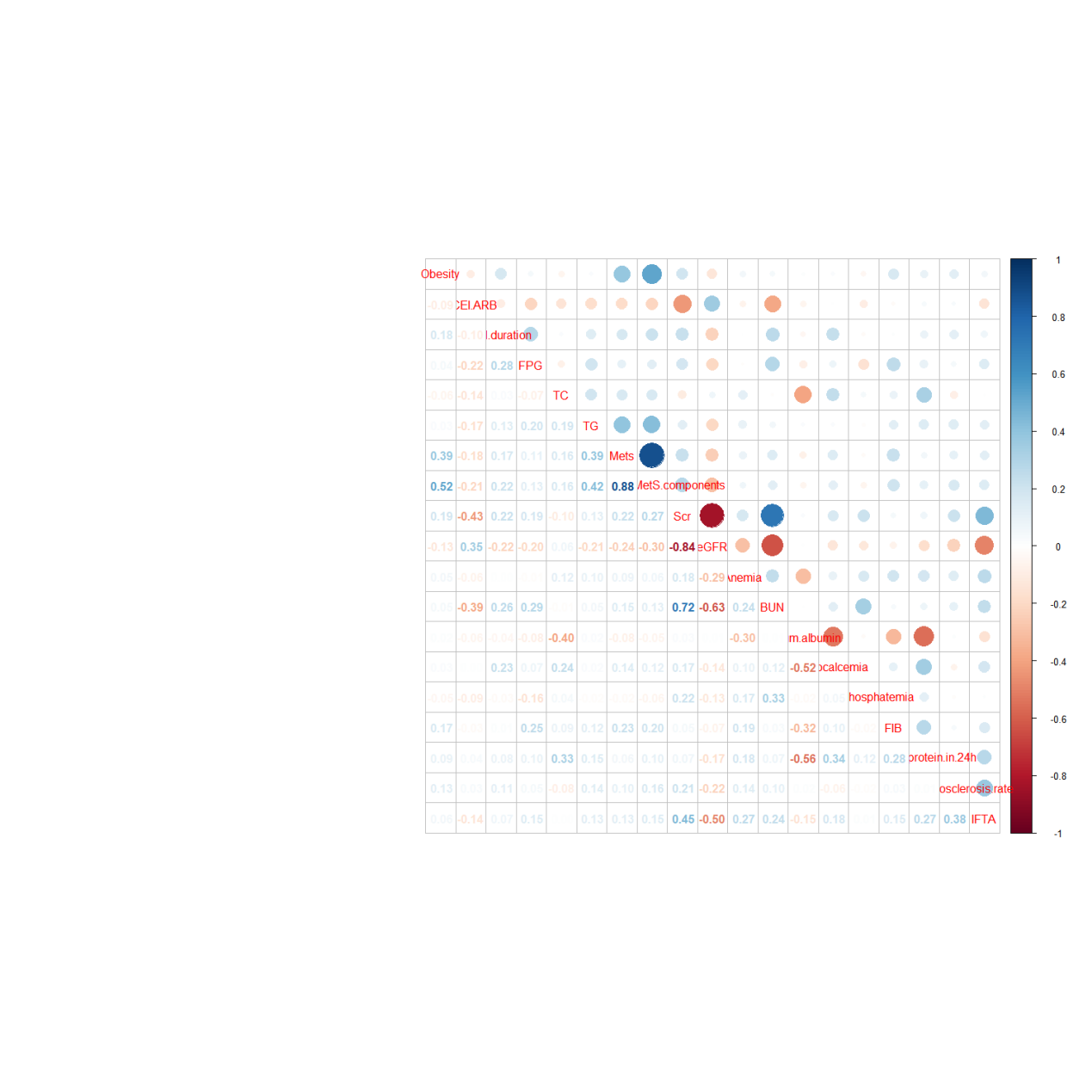


**Figure S3.** Correlation analysis among variables selected through univariate regression analysis.

**
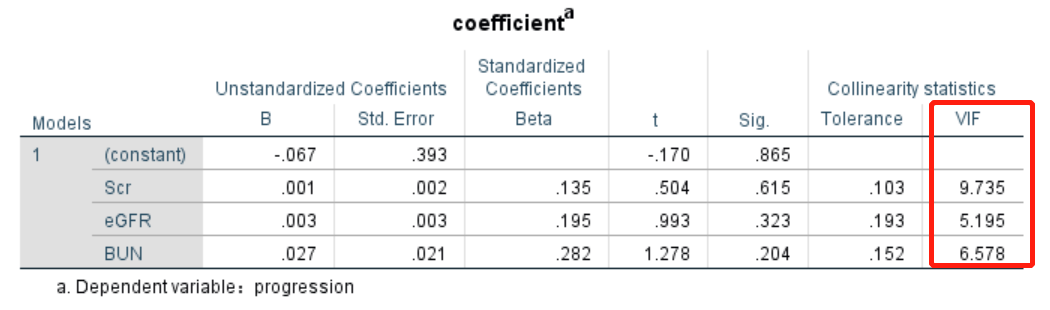
**

**Figure S4.** Collinearity statistics among Scr, eGFR and BUN.


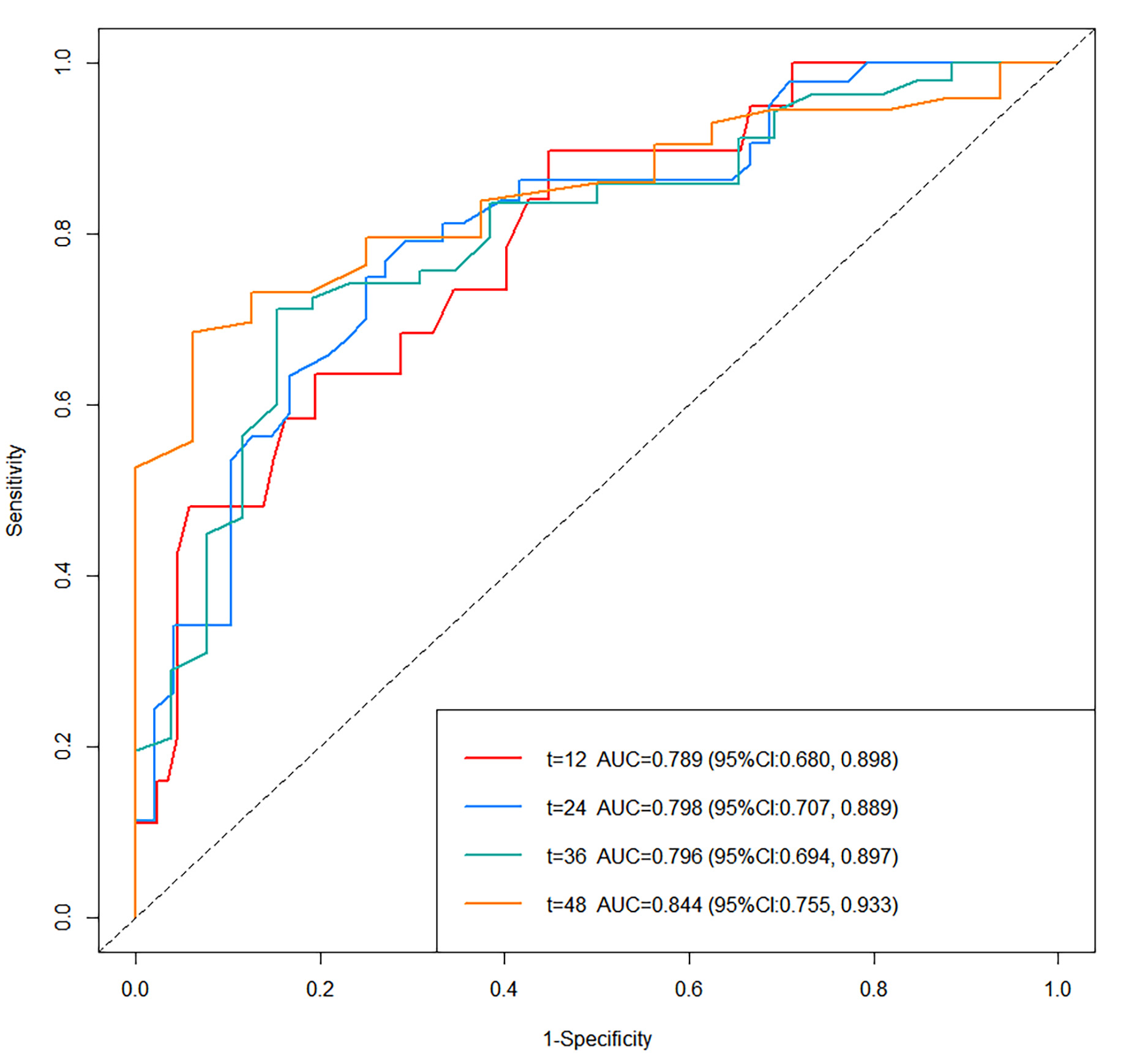


**Figure S5** Time-dependent ROC curves for the reconstructed model by redefining MetS.


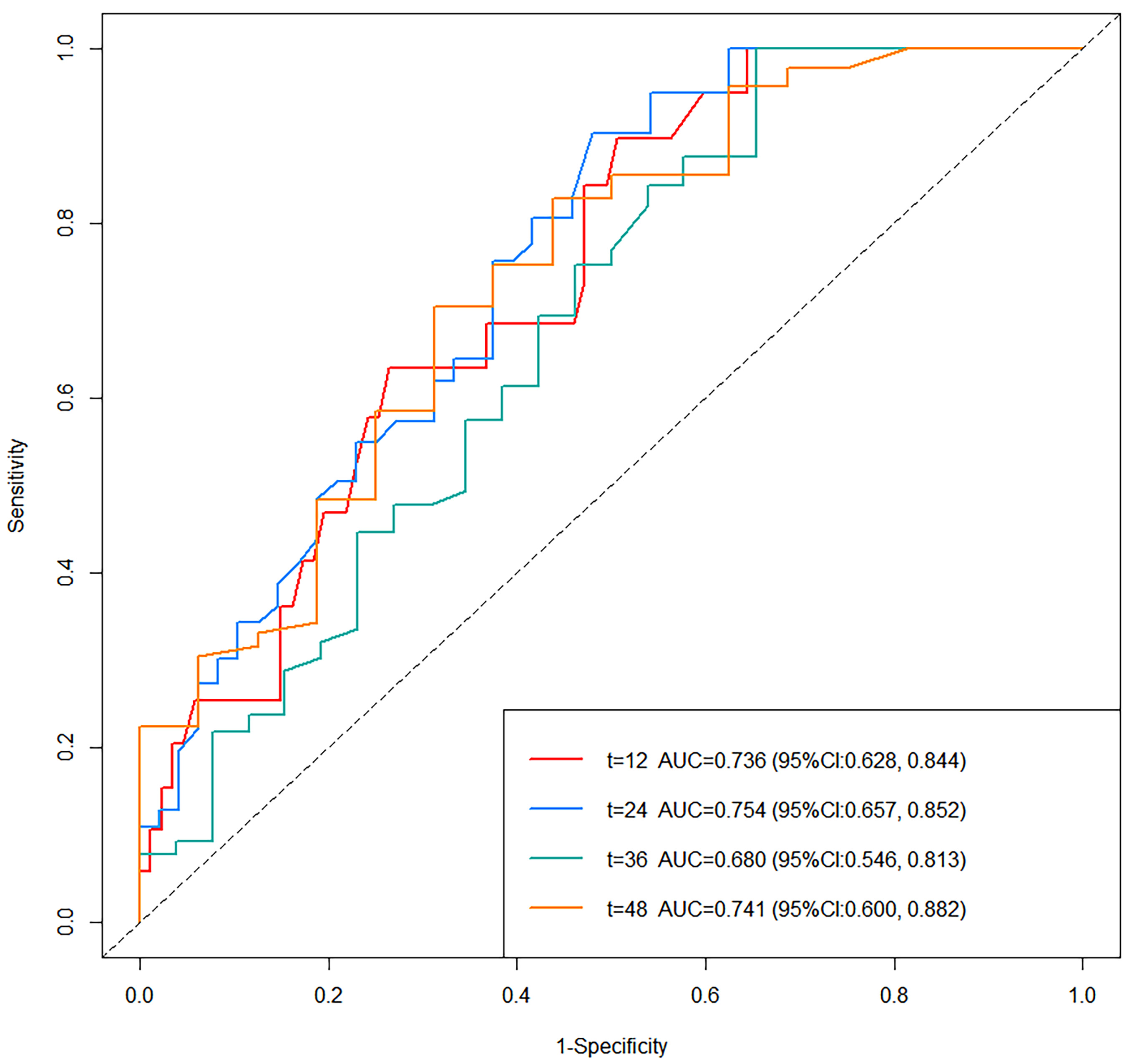


**Figure S6** Time-dependent ROC curves for the model constructed by factors from the previously validated model.

**Table S1.** The proportion of the missing data.

| **Variables** | **Full names of abbreviations** | **Missingness rates** |
| --- | --- | --- |
| PCT | Procalcitonin | 25.95% |
| C1q | Complement component 1q | 22.78% |
| C4 | Complement component 4 | 15.19% |
| UACR | Urinary albumin to-creatinine ratio | 14.56% |
| C3 | Complement component 3 | 13.92% |
| HbA1c | Glycosylated hemoglobin, type A1c | 11.39% |
| PTH | Parathyroid hormone | 11.39% |
| CRP | C-reactive protein | 1.27% |

**Table S2** The cutoff points and selection criteria for the variables.

| **Variables** | **cutoff points** | **selection criteria** |
| --- | --- | --- |
| Gender | Male/Female | - |
| Smoking | Yes/No | Yes:Current smokers or former smokers |
| DM duration | - | Duration from diabetes diagnosis to DN diagnosis. |
| BMI | - | Weight (kg)/Height (m)^2^ |
| Obesity | Yes/No | Obesity was defined as BMI>25.0 kg/m^2^ according to the diagnostic criteria for MetS proposed by the Chinese Diabetes Society (CDS) in 2004. |
| HbA1c | - | Reference range: 4.0%–6.0% |
| FPG | - | Reference range: 3.9–5.6 mmol/L |
| TC | - | Reference range: <5.2 mmol/L |
| TG | - | Reference range: <1.7 mmol/L |
| HDL-C | - | Reference range: >1.03 mmol/L in men; >1.29 mmol/L in women |
| LDL-C | - | Reference range: <3.4 mmol/L |
| Scr | - | Reference range: 62–106 μmol/L in men; 44–97 μmol/L in women |
| eGFR | - | G1: ≥90ml/min/1.73m^2^  G2: 60-89ml/min/1.73m^2^  G3a: 45-59ml/min/1.73m^2^  G3b: 30-44ml/min/1.73m^2^  G4: 15-29ml/min/1.73m^2^  G4: <15ml/min/1.73m^2^ |
| BUN | - | Reference range: 3.1-8.0mmol/L |
| Serum albumin | - | Reference range:40.0-55.0g/L |
| urinary protein in 24h | - | Reference range:<150 mg/24h |
| FIB | - | Reference range: 2-4g/L |
| Use of ACEI/ARB | Yes/No | Yes: ACEI/ARB therapy was initiated either prior to or upon diagnosis of biopsy-confirmed diabetic kidney disease and maintained until study endpoint or development of contraindications. |
| Hypertension | Yes/No | Hypertension was defined per WHO criteria: SBP ≥140 mmHg and/or DBP ≥90 mmHg confirmed on two separate visits, or the diagnosis have been verified by use of anti-hypertension medications. |
| Cardiovascular disease | Yes/No | Cardiovascular disease was defined as a previous diagnosis of heart failure, myocardial infarction, valvular heart disease, percutaneous coronary intervention, or bypass grafting. |
| Cerebrovascular disease | Yes/No | Cerebrovascular disease was defined as a previous diagnosis of cerebral infarction, cerebral hemorrhage, or stroke. |
| Anemia | Yes/No | Anemia is defined as hemoglobin levels <130 g/L in men and <120 g/L in women according to WHO diagnostic criteria. |

**Table S2** (continued)

| **Variables** | **cutoff points** | **selection criteria** |
| --- | --- | --- |
| Hyperuricemia | Yes/No | Hyperuricemia is defined as serum uric acid levels exceeding 6.8 mg/dL (≈420 μmol/L) according to American College of Rheumatology criteria. |
| Hypocalcemia | Yes/No | Hypocalcemia is defined as a corrected total serum calcium level <2.20 mmol/L or ionized calcium <1.12 mmol/L according to American Endocrine Society criteria. |
| Hyperphosphatemia | Yes/No | Hyperphosphatemia is defined as fasting serum phosphate >1.45 mmol/L according to KDIGO 2023 CKD-MBD Guideline. |
| MetS | Yes/No | Metabolic syndrome was defined according to the modified criteria of the National Cholesterol Education Program Adult Treatment Panel Third Report (ATP III). |
| Number of MetS components | 1/2/3/4/5 | The MetS components including central obesity or abdominal obesity, elevated blood pressure, increased plasma triglycerides or treated dyslipidemia, low fasting HDL-C and elevated fasting glucose or use of anti-diabetic medication.  1 component: As all participants had DN, each individual met at least one component of MetS.  2 components: Patients had two components of MetS, including diabetes.  3 components: Patients had three components of MetS, including diabetes.  4 components: Patients had four components of MetS, including diabetes.  5 components: Patients had all the components of MetS. |
| Glomerulosclerosis rate | - | Glomerulosclerosis rate (%) = [Number of sclerotic glomeruli (global + segmental) / Total glomeruli] × 100% |
| K-W nodules | Yes/No | Characteristic nodular lesions in DN, resulting from profound mesangial matrix accumulation, display concentric laminations under periodic acid-Schiff (PAS) and periodic acid-silver methenamine (PASM) stains. |
| IFTA scores | - | According to the criteria proposed by Renal Pathology Society. |
| Renal interstitial inflammation scores | - | According to the criteria proposed by Renal Pathology Society. |
| Vascular scores | - | According to the criteria proposed by Renal Pathology Society. |
| Abbreviations: DN: diabetic nephropathy; DM: diabetes mellitus; BMI: body mass index; HbA1c: glycosylated hemoglobin, type A1c; FPG: fasting blood glucose; TC: total cholesterol; TG: triglycerides; HDL-C: high density lipoprotein cholesterol; LDL-C: low density lipoprotein cholesterol; Scr: serum creatinine; eGFR: estimated glomerular filtration rate; BUN: blood urea nitrogen; FIB: fibrinogen; ACEI: angiotensin converting enzyme inhibitors; ARB: angiotensin receptor antagonist; MetS: metabolic syndrome; IFTA: interstitial fibrosis and tubular atrophy; WHO: World Health Organization | | |

**Table S3** Univariate Cox regression analysis of MetS and the number of MetS components defined by 2004 CDS criteria.

|  | **Univariate regression analysis** | |
| --- | --- | --- |
|  | **HR (95%CI)** | ***P* value** |
| MetS | 2.803 (1.557, 5.047) | 0.001^*^ |
| Number of MetS components  (compared with 1 or 2 components) |  |  |
| 3 components | 2.695 (1.459-4.980) | 0.002^*^ |
| 4 components | 3.089 (1.510-6.317) | 0.002^*^ |
| ^*^*P* value<0.05.  Abbreviations: MetS: metabolic syndrome; CDS: Chinese Diabetes Society; HR:Hazard Ratio. | | |

**Table S4** Variables selection in sensitivity analysis using LASSO regression analysis and multivariate Cox regression analysis.

| **Variables** | **LASSO regression analysis**  **λ (1-SE) = 0.077** |  | **Multivariate regression analysis** | |
| --- | --- | --- | --- | --- |
|  | **Coefficients** |  | **HR (95%CI)** | ***P* value** |
| Use of ACEI/ARB | -0.367 |  | 0.536 (0.303-0.950) | 0.033^*^ |
| DM duration | 0.002 |  | - | - |
| FPG | 0.049 |  | 1.095 (1.014-1.182) | 0.020^*^ |
| Number of MetS components  (compared with 1 or 2 components including DM) | 0.292 |  |  |  |
| 3 components |  |  | 3.228 (1.627-6.405) | 0.001^*^ |
| 4 components |  |  | 2.787 (1.239-6.268) | 0.013^*^ |
| eGFR | -0.001 |  | - | - |
| Serum albumin | -0.020 |  | - | - |
| Hyperphosphatemia | 0.198 |  | 2.204 (1.174-4.136) | 0.014^*^ |
| FIB | 0.059 |  | 1.142 (1.004-1.298) | 0.043^*^ |
| Urinary protein in 24h | 0.024 |  | 1.088 (1.008-1.174) | 0.030^*^ |
| Glomerulosclerosis rate | 0.003 |  | - | - |
| ^*^*P* value<0.05.  Abbreviations: LASSO; Least absolute shrinkage and selection operator; DN: diabetic nephropathy; DM: diabetes mellitus; FPG: fasting blood glucose; eGFR: estimated glomerular filtration rate; FIB: fibrinogen; ACEI: angiotensin converting enzyme inhibitors; ARB: angiotensin receptor antagonist; MetS: metabolic syndrome; HR: Hazard Ratio; SE: standard error. | | | | |
